# Similar results with quadrupled semitendinosus and semitendinosus‐gracilis graft in anterior cruciate ligament reconstruction: A randomised controlled trial with 2‐year follow‐up

**DOI:** 10.1002/jeo2.70399

**Published:** 2025-09-24

**Authors:** Juan P. Martinez‐Cano, Alejandro Gallego, Janio Cuadros, Laura Ibarra, Fernando M. Mejía, María V. Velasquez‐Hammerle, Alfredo Martinez‐Rondanelli

**Affiliations:** ^1^ Fundación Valle del Lili, Departamento de Ortopedia Cali Colombia; ^2^ Universidad Icesi Cali Colombia; ^3^ Fundación Valle del Lili, Centro de Investigaciones Clínicas (CIC) Cali Colombia; ^4^ Musculoskeletal Translational Innovation Initiative, Beth Israel Deaconess Medical Center Department of Orthopaedic Surgery Harvard Medical School Boston USA

**Keywords:** anterior cruciate ligament injuries, anterior cruciate ligament reconstruction, knee joint, muscle strength, patient outcome assessment

## Abstract

**Purpose:**

to compare quadrupled semitendinosus (STx4) with semitendinosus‐gracilis graft in anterior cruciate ligament (ACL) reconstruction surgery.

**Methods:**

Parallel randomised controlled trial with two groups of treatment (*n* = 42) in primary ACL reconstruction surgery. Follow‐up during 2 years after surgery with visits at 3, 6, 12 and 24 months. Primary outcome: quadriceps and hamstrings strength in newtons (N). Secondary outcomes: ACL re‐rupture, additional surgeries, return to sport and patient‐reported outcomes (PROMs): knee injury and osteoarthritis outcome score (KOOS), International Knee Documentation Committee (IKDC) and Tegner‐Lysholm.

**Results:**

There was 1/21 ACL re‐rupture for STx4 and 2/21 for the control group (*p* = 0.9). One additional surgery for each group: ACL revision (control group) and meniscectomy (STx4). There were no other complications and no differences in PROMs between grafts, except for Tegner‐Lysholm at 3 months, that favoured the STx4 group (76.0, confidence interval [CI]: 56.0–86.0 vs. 85.0, CI: 77.0–93.0), *p* = 0.04. The median surgery satisfaction was good in both groups STx4 (95%, interquartile range (IQR): 90%–98%) and ST‐G (98%, IQR: 95%–100%) (*p* = 0.13). Return to sport was 90% (*n* = 19) for the STx4 group and 81% (*n* = 17) for the ST‐G group (*p* = 0.37). Quadriceps strength recovered sooner (6 months) than hamstrings (24 months) in both groups. There were no statistically significant differences in strength between STx4 and ST‐G at final follow‐up (hamstrings in flexion [mean ± SD]: 173 ± 53 N vs. 166 ± 51 N, *p* = 0.7; hamstrings in extension: 204 ± 61 N vs. 199 ± 64 N, *p* = 0.8; quadriceps in flexion: 217 ± 42 N vs. 209 ± 47 N, *p* = 0.6; quadriceps in extension: 193 ± 51 N vs. 191 ± 46 N, *p* = 0.9).

**Conclusions:**

This study found no statistically significant differences at 2 years between a STx4 and a ST‐G graft configuration for primary ACL reconstruction regarding strength, PROMs, return to sport, failure rates and complications; larger studies are required to confirm noninferiority. Clinical Trials number: NCT03433170.

**Level of Evidence:**

Level I.

**Abbreviations**
ACL: anterior cruciate ligament
ACLr: anterior cruciate ligament reconstruction
BMI: body mass index
CI: confidence interval
ICC: intraclass correlation coefficients
IKDC: International Knee Documentation Committee
IQR: interquartile range
KLT: knee laxity tester
KOOS: knee injury and osteoarthritis outcome score
KSS: knee society score
MRI: magnetic resonance imaging
N: newtons
PASS: patient acceptable symptom state
PROMs: patient‐reported outcomes
SD: standard deviation
STx4: quadrupled semitendinosus
ST‐G: semitendinosus‐gracilis group

## INTRODUCTION

Anterior cruciate ligament reconstruction (ACLR) is a commonly performed knee surgery [12, 27]. Multiple techniques have been described for ACLR, with different graft options [1, 11, 19, 22]. The quadrupled hamstrings graft has shown the highest load to failure and stiffness [5]. However, a recent randomised controlled trial has shown hamstrings failure of 11.2% in high‐risk patients (pivot‐shift ≥2, participation in pivoting sport and generalised ligamentous laxity) [28]. Hamstrings graft has risen to become one of the most frequently used graft in the past years [17, 24, 33], possibly explained by the fact that they are easier to harvest and are associated with less donor‐site morbidity and postoperative quadriceps weakness [3, 4].

Several variations for the hamstrings graft have been described, in which a quadruple strand configuration is obtained with either a double strand semitendinosus plus double strand gracilis tendon graft, or using only the semitendinosus tendon and folding it onto itself into a quadruple strand configuration [21]. The latter one is a shorter tendon due to its quadruple configuration, with the potential of a greater diameter and allowing the preservation of the gracilis.

The quadrupled semitendinosus graft has been compared to the patellar bone‐tendon‐bone graft, demonstrating good outcomes and less anterior knee pain [2]. With regards to this graft, instead of using the semitendinosus and the gracilis, it has been proposed that preserving the gracilis has a lower impact on strength and function loss to the knee [31]. However, outcomes have been mixed with regards to the theoretical advantage in muscle strength when comparing the quadrupled semitendinosus to the semitendinosus and gracilis graft configurations. Roger et al., performed a randomised controlled trial and didn't find statistically significant differences among both groups for quadriceps or hamstring strength (*n* = 60) [29]. On the other hand, Kouloumentas et al. did report superior knee flexion strength for the quadrupled semitendinosus group in their randomised controlled trial (*n* = 90) [14]. Both studies were published posteriorly to the start of our clinical trial.

The principal purpose of this study was to compare ACL reconstruction surgery with quadrupled semitendinosus graft compared to semitendinosus‐gracilis graft. We hypothesised the quadrupled semitendinosus graft group would show superior hamstrings strength in follow‐up with similar complications and PROMs.

## METHODS

### Study design

This randomised controlled parallel clinical trial was performed in a university hospital in *Cali, Colombia*. It was approved by the ethics committee at the research department in the institution.

### Study subjects

**Figure 1:**
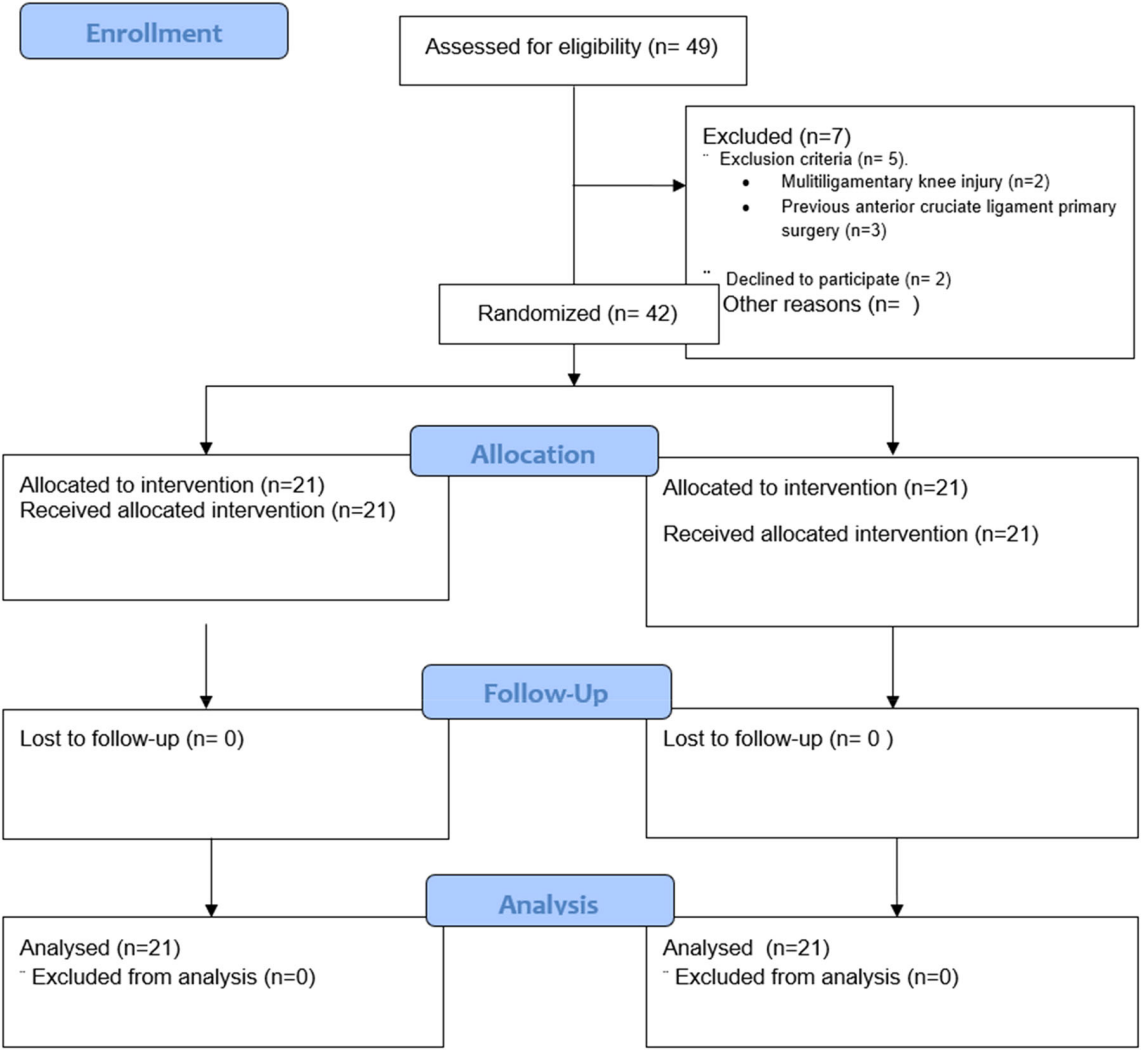
Flow diagram of patients in the study.

Patients from the institution, with a complete ACL tear diagnosed clinically and confirmed with magnetic resonance imaging (MRI), over 14 years old, both sexes, and willing to participate in the study were included. All patients were treated surgically between January 2018 and June 2020. Exclusion criteria included multi‐ligament lesions and ACL revision cases. Figure 1 shows the flow diagram of participants.

Planned total follow‐up was 2 years, with preoperative and postoperative 3, 6, 12 and 24 months follow‐up timepoints. Forty‐two patients were included in the study, of which 100% were followed up for 2 years. Patients were distributed in two groups with an allocation ratio of 1:1 (quadrupled semitendinosus/semitendinosus‐gracilis), each with 21 participants.

Randomisation was done by an external member of the research centre, with a software that distributed random numbers in blocks of 10 patients. Sealed envelopes with the graft types assigned were distributed as indicated by the software and opened right at the start of the surgical procedure once the patient had been induced under anaesthesia. All patients were treated by two surgeons from the same institution, experts in both surgical techniques.

Patients remained blinded to the intervention allocation until after the 2 year follow up mark. No surgical description data that could reveal the type of hamstrings graft assigned were included, to maintain blinding. Physicians evaluating PROMs and other variables measured included in the outcomes of interest for the study were blind to the surgical treatment as well.

### Outcome measures

In the initial preoperative visit, demographic data were obtained, such as age, sex, side injured, weight, height and injury characteristics. Participants were required to attend postoperative clinic visits at 3‐, 6‐, 12‐ and 24‐month timepoints. Primary and secondary outcomes were evaluated in each visit.

The primary outcome in this study was knee flexion and extension strength. This was measured in newtons (N) in four different positions with a hand‐held dynamometer (Lafayette, Fabrication Enterprises). Knee flexion was evaluated with the patient in supine position, with the knee flexed to 90 degrees and in full extension, against resistance for 3 s, registering the peak strength value. Similarly, knee extension was evaluated with the patient in prone position, with the knee in full extension and at 90 degrees of flexion. Each measurement was repeated three times, and the highest value was recorded.

Secondary outcomes included re‐tear, evaluated with clinical tests: Lachman and anterior drawer. Additionally, the KLT (Knee Laxity Tester, Storz) device was used to measure anterior tibial displacement in millimetres during Lachman test. If a re‐tear was suspected, MRI would be used to confirm. Other secondary outcomes included PROMs such as knee injury and osteoarthritis outcome score (KOOS), International Knee Documentation Committee (IKDC), Tegner, and Lysholm scales, complications, re‐interventions, return to preinjury level of activity and patient self‐reported satisfaction with the procedure from 0% to 100%.

### Surgical technique

Conventional knee arthroscopy was performed, with parapatellar medial and lateral portals. A femoral tunnel from the medial portal was performed with cortical femoral fixation (XO button, Conmed) and interference screw in the tibia (Xtralok, Conmed) with the knee in 15 degrees of flexion and neutral rotation was performed. A pneumatic tourniquet was used only if bleeding obscured visibility significantly. Hamstring graft harvest was performed with an incision of 2–3 cm over the pes anserine, releasing either both tendons or only the semitendinosus from their tibial insertion, depending on the randomisation. Harvest was completed with a closed tendon stripper. Tendons were then irrigated with saline and vancomycin 1 g to prevent infections [6]. Grafts were prepared in a four‐strand configuration: Either the semitendinosus quadruple or double semitendinosus and double gracilis together. In case of obtaining a graft size inferior to 8 mm when using a semitendinosus and gracilis graft, the semitendinosus was tripled to achieve a higher diameter graft as data has shown increased re‐tear incidence with grafts smaller than 8 mm [20]. Supporting Information S1: File S1 includes three figures regarding the surgical technique.

### Statistics

Sample size was calculated to achieve 80% power, with an alpha error of 5%, to estimate a mean difference of at least 50% in hamstrings strength at 6 months between both groups in knee flexion. This calculation gave 19 patients in each group, which was increased to 21 per group, in case of possible lost to follow‐up patients. Averages and confidence interval of 95% (95% CI) were used for normally distributed variables, while median and interquartile range were used for nonnormally distributed variables. Student *t* test was the parametric test used, while the nonparametric tests were chi‐squared and Fisher's exact test.

A linear mixed model with repeated measures [32] was performed to analyse clinically relevant outcomes, assessed at 3, 6, 12 and 24 months of follow‐up. The model was adjusted for the initial type of surgery, incorporating random effects to account for between‐subject variability, and treatment by linear time interaction models.

All statistical analysis was performed using STATA software (version 18, Stata Corp.). *p*‐value < 0.05 was considered statistically significant.

## RESULTS

A total of 42 patients were included in this study, distributed in two groups of 21 patients each. The *experimental* group was the quadrupled semitendinosus (STx4), and the *control* group was the semitendinosus‐gracilis group (ST‐G). Follow up of 2 years was achieved for 100% of patients. Table 1, shows the baseline characteristics of both groups.

**Table 1:** Baseline characteristics for both groups.

Variables	Group, mean (95% CI)^a^	
	Control, *n* = 21	STX4, *n* = 21
Sex		
Female	3 (14.3%)	6 (28.6%)
Male	18 (85.7%)	15 (71.4%)
Age	29 (22.0–37.0)	23 (18.0–27.0)
Height	169.3 ± 6.9	168.6 ± 10.9
Weight	77.2 ± 12.6	71.1 ± 15.9
BMI	26.9 ± 3.8	24.8 ± 4.0
Side		
Left	13 (61.9%)	13 (61.9%)
Right	8 (38.1%)	8 (38.1%)
Injury aetiology		
Soccer	11 (52.4%)	14 (66.7%)
Car crash	1 (4.8%)	3 (14.3%)
Walking	2 (9.5%)	1 (4.8%)
Jogging	1 (4.8%)	0 (0.0%)
Fall	1 (4.8%)	0 (0.0%)
Weight lifting	1 (4.8%)	0 (0.0%)
Fall from their own height	1 (4.8%)	0 (0.0%)
Work‐related accident	1 (4.8%)	0 (0.0%)
Other sports	1 (4.8%)	0 (0.0%)
Cheerleading	1 (4.8%)	0 (0.0%)
Cycling	0 (0.0%)	1 (4.8%)
Dance	0 (0.0%)	1 (4.8%)
Skiing	0 (0.0%)	1 (4.8%)
Pivot shift		
0	1 (4.8%)	1 (4.8%)
1	8 (38.1%)	9 (42.9%)
2	10 (47.6%)	11 (52.4%)
3	2 (9.5%)	0 (0.0%)

Abbreviations: BMI, body mass index; CI, confidence interval.

^a^ Data presented as mean and standard deviation (SD) or median and interquartile range.

Intraoperative variables for both groups are presented in Table 2. Associated cartilage and meniscal injuries were similar in both groups. Surgical time was slightly longer in STx4 group (8 min) with no statistically significant difference. Mean graft diameter were similar between STx4 and ST‐G groups (8.85 ± 0.67 mm vs. 8.54 ± 1.12 mm), with no statistically significant difference. When adjusting for those cases in which the semitendinosus was tripled in the control group, mean graft diameter increased to 9.0 ± 0.5 mm in that group. The median self‐reported satisfaction in STx4 group was 95% (interquartile range [IQR]: 90%–98%) and 98% (IQR: 95%–100%) in the ST‐G group, with no significant difference between groups (*p* = 0.13).

**Table 2:** Intraoperative variables.^a^

	Group, mean (95% CI)		*p* value^b^
	Control, *N* = 21	STx4, *N* = 21	
Surgical time, minutes	74.0 (67.0–86.0)	82.0 (76.0–92.0)	0.066
Tourniquet use	7 (33.3%)	6 (28.6%)	0.9
Graft diameter	8.54 ± 1.116	8.85 ± 0.67	0.284
Final graft diameter	9.0 ± 0.5	‐	NA
STx4 graft length	‐	7.1 ± 0.7	NA
Partial preservation of ACL	4.3 ± 9.4	10.2 ± 15.8	0.13
Meniscal injury			0.9
No injury	5 (23.8%)	3 (14.3%)	
Medial meniscus	3 (14.3%)	4 (19.0%)	
Lateral meniscus	8 (38.1%)	9 (42.9%)	
Both	5 (23.8%)	5 (23.8%)	
Suture			0.9
Medial meniscus	3 (14.3%)	4 (19.0%)	
Lateral meniscus	7 (33.3%)	7 (33.3%)	
Both	2 (9.5%)	3 (14.3%)	
Meniscectomy			0.2
Medial meniscus	1 (4.8%)	2 (9.5%)	
Lateral meniscus	2 (9.5%)	3 (14.3%)	
Both	3 (14.3%)	0 (0.0%)	
Acute cartilage lesion in lateral compartment	1 (4.8%)	2 (9.5%)	0.9
Acute cartilage lesion in medial compartment	3 (14.3%)	1 (4.8%)	0.6
Acute patellar cartilage lesion	3 (14.3%)	3 (14.3%)	0.9
Acute trochlear cartilage lesion	1 (4.8%)	0 (0.0%)	0.9
Chronic cartilage lesion lateral compartment	0 (0.0%)	3 (14.3%)	0.2
Chronic cartilage lesion medial compartment	3 (14.3%)	2 (9.5%)	0.9
Chronic patellar cartilage lesion	1 (4.8%)	3 (14.3%)	0.6
Chronic trochlear cartilage lesion	1 (4.8%)	0 (0.0%)	0.9

Abbreviations: ACL, anterior cruciate ligament; CI, confidence interval.

^a^ Data presented as means (standard deviation), median (interquartile ranges), *N* (%) (*p* < 0.05).

^b^ *T*‐test and Mann–Whitney *U* test for parametric variables, chi‐squared and Fisher’ exact test for nonparametric variables.

Peak strength for quadriceps and hamstrings with the knee in extension and at 90 degrees flexion can be seen in Table 3. Although there was a tendency to higher strength in the STx4 group, there was no statistically significant difference between groups at any timepoint.

**Table 3:** Peak muscle strength in newtons (N) throughout different measure points.^a^

	Group, mean (95% CI)		Difference in means (95% CI)	*p* value^b^
	Control *N* = 21	STx4, *N* = 21		
Hamstrings in flexion				
Pre‐operative	118.7 ± 29.0	131.3 ± 32.1	−13 (−32 to 6.5)	0.2
3 months	108.4 (102.4–121.9)	113.6 (99.3–126.4)	−3.0 (−23 to 17)	0.8
6 months	127.1 ± 36.7	140.4 ± 38.8	−13 (−37 to 10)	0.3
12 months	123.0 (103.5–178.8)	122.8 (112.5–153.9)	−7.9 (−38 to 22)	0.6
24 months	165.8 ± 50.8	172.7 ± 52.9	−6.9 (−40 to 26)	0.7
Hamstrings in extension				
Pre‐operative	129.9 ± 37.4	146.4 ± 44.3	−16 (−42 to 9.2)	0.2
3 months	139.1 (131.6–146.3)	135.7 (122.7–159.9)	−18 (−22 to 18)	0.9
6 months	168.0 ± 38.9	171.6 ± 46.1	−3.7 (−30 to 23)	0.8
12 months	165.8 (140.9–211.8)	153.7 (141.5–188.5)	1.7 (−30 to 33)	0.9
24 months	198.6 ± 63.6	203.6 ± 61.3	−5.0 (−45 to 35)	0.8
Quadriceps in flexion				
Pre‐operative	142.4 ± 43.3	149.0 ± 45.7	−6.7 (−34 to 21)	0.6
3 months	140.6 ± 36.4	151.4 ± 47.0	−11 (−37 to 15)	0.4
6 months	155.6 ± 41.3	178.4 ± 50.1	−23 (−51 to 5.9)	0.12
12 months	176.7 ± 49.3	180.2 ± 46.0	−3.5 (−33 to 26)	0.8
24 months	208.7 ± 47.0	216.6 ± 41.7	−7.9 (−37 to 21)	0.6
Quadriceps in extension				
Pre‐operative	129.2 ± 43.7	140.5 ± 48.9	−11 (−40 to 18)	0.4
3 months	140.9 ± 34.9	156.3 ± 46.9	−15 (−41 to 10)	0.2
6 months	163.2 ± 38.3	170.3 ± 45.8	−7.1 (−33 to 19)	0.6
12 months	178.6 ± 45.8	166.4 ± 46.2	12 (−17 to 41)	0.4
24 months	190.6 ± 45.7	193.4 ± 51.4	−2.8 (−34 to 28)	0.9

Abbreviation: CI, confidence interval.

^a^ Data presented as means (standard deviation) or median (interquartile range) (*p* < 0.05).

^b^ *T*‐test and Mann–Whitney *U*‐test for parametric variables.

Table 4 shows PROMs results and values obtained when testing laxity with the KLT device. There was a statistically significant difference only for the Tegner Lysholm scale at the 3‐month postop follow‐up, favouring the STx4 group (median 76.0, IQR: 56–86 points vs. 85.0, IQR: 77–94 points, *p* = 0.039). With regards to laxity, it was higher for the STx4 group at the 12‐month postop mark (anterior displacement of 7.43 ± 1.66 mm vs. 6.42 ± 1.46 mm, *p* = 0.04), with no statistically significant difference at the 2‐year follow‐up timepoint (ST‐G: 6.76 ± 1.70 mm and STx4: 6.82 ± 1.72 mm, *p* = 0.92).

**Table 4:** Patient‐reported outcome measures (PROMs) and stability in both groups.^a^

	Type of surgery, mean (95% CI)		Difference in means (95% CI)	*p*‐Value^b^
	Control, *N* = 21	STX4, *N* = 21		
IKDC				
Pre‐operative	43.8 ± 16.8	49.7 ± 16.2	−5.8 (−16 to 4.5)	0.3
3 months	62.8 ± 16.6	66.0 ± 14.5	−3.1 (−13 to 6.6)	0.5
6 months	76.2 ± 13.0	79.8 ± 14.9	−3.6 (−12 to 5.1)	0.4
12 months	85.1 (72.4–93.1)	88.5 (79.3–95.4)	−4.7 (−17 to 7.7)	0.4
24 months	92.0 (75.3–96.9)	92.0 (88.5–98.9)	−4.6 (−13 to 3.4)	0.3
Tegner‐Lysholm				
Pre‐operative	54.2 ± 22.8	54.7 ± 22.7	−0.43 (−15 to 14)	0.9
3 months	76.0 (56.0–86.0)	85.0 (77.0–93.0)	−12 (−23 to −0.62)	0.039
6 months	90.0 (78.0–95.0)	92.0 (90.0–95.0)	−2.2 (−11 to 7.1)	0.6
12 months	92.0 (80.0–100.0)	95.0 (90.0–99.0)	−3.5 (−14 to 7.1)	0.5
24 months	95.0 (83.8–100.0)	95.0 (92.0–100.0)	−2.9 (−10 to 4.5)	0.4
KOOS				
Pre‐operative	64.1 ± 15.2	63.2 ± 19.3	0.87 (−10 to 12)	0.9
3 months	79.8 (69.0–85.1)	83.9 (79.2–92.1)	−5.3 (−12 to 1.8)	0.14
6 months	88.1 (78.6–94.0)	92.3 (86.3–95.2)	−3.9 (−9.6 to 1.8)	0.2
12 months	91.7 (86.3–96.3)	95.2 (92.3–97.6)	−4.4 (−12 to 3.3)	0.3
24 months	95.8 (92.0–99.1)	95.8 (92.3–98.2)	−0.65 (−6.6 to 5.3)	0.8
Anterior tibia displacement				
Pre‐operative	8.74 ± 1.82	9.53 ± 1.69	−0.79 (−2.08 to 0.51)	0.22
3 months	6.54 ± 1.11	6.62 ± 1.4	−0.08 (−0.975 to 0.815)	0.85
6 months	6.43 ± 1.62	7.02 ± 1.70	−0.59 (−1.68 to 0.51)	0.28
12 months	6.42 ± 1.66	7.43 ± 1.46	−1.01 (−2.00 to 0.025)	0.04
24 months	6.76 ± 1.70	6.82 ± 1.72	−.051 (−1.15 to 1.05)	0.92

*Note*: *p*‐Values in bold mean there were statistically significant results.Abbreviations: CI, confidence interval; IKDC, International Knee Documentation Committee; KOOS, knee injury and osteoarthritis outcome score.

^a^ Data presented in means (standard deviation) or medians (interquartile range) (*p* < 0.05).

^b^ *T*‐test and the Mann–Whitney *U*‐test were used for parametric variables.

**Figure 2:**
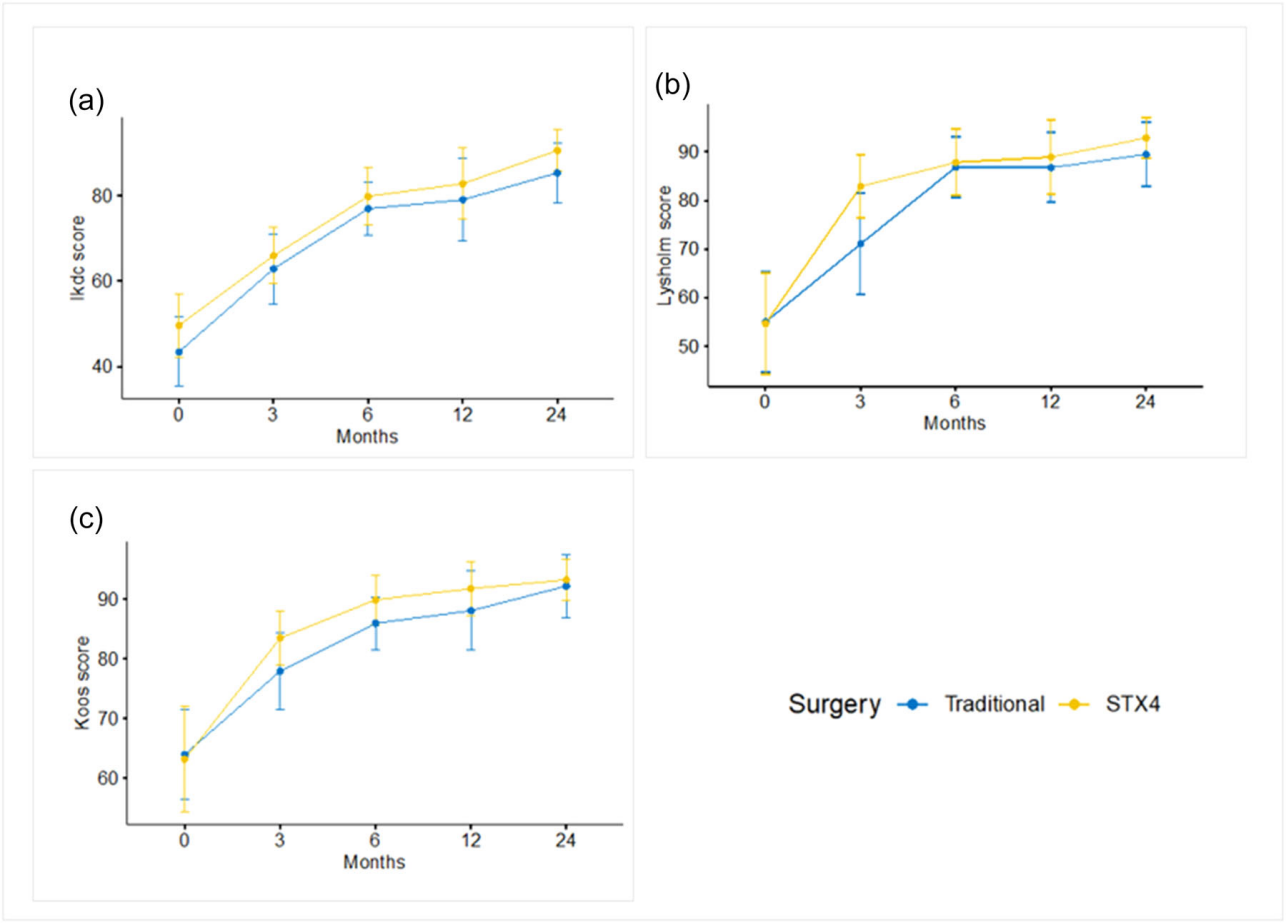
Patient‐reported outcomes (PROMs). (a) International Knee Documentation Committee (IKDC) scores. (b) Tegner Lysholm scores. (c) Knee injury and osteoarthritis outcome score (KOOS) scores.

**Figure 3:**
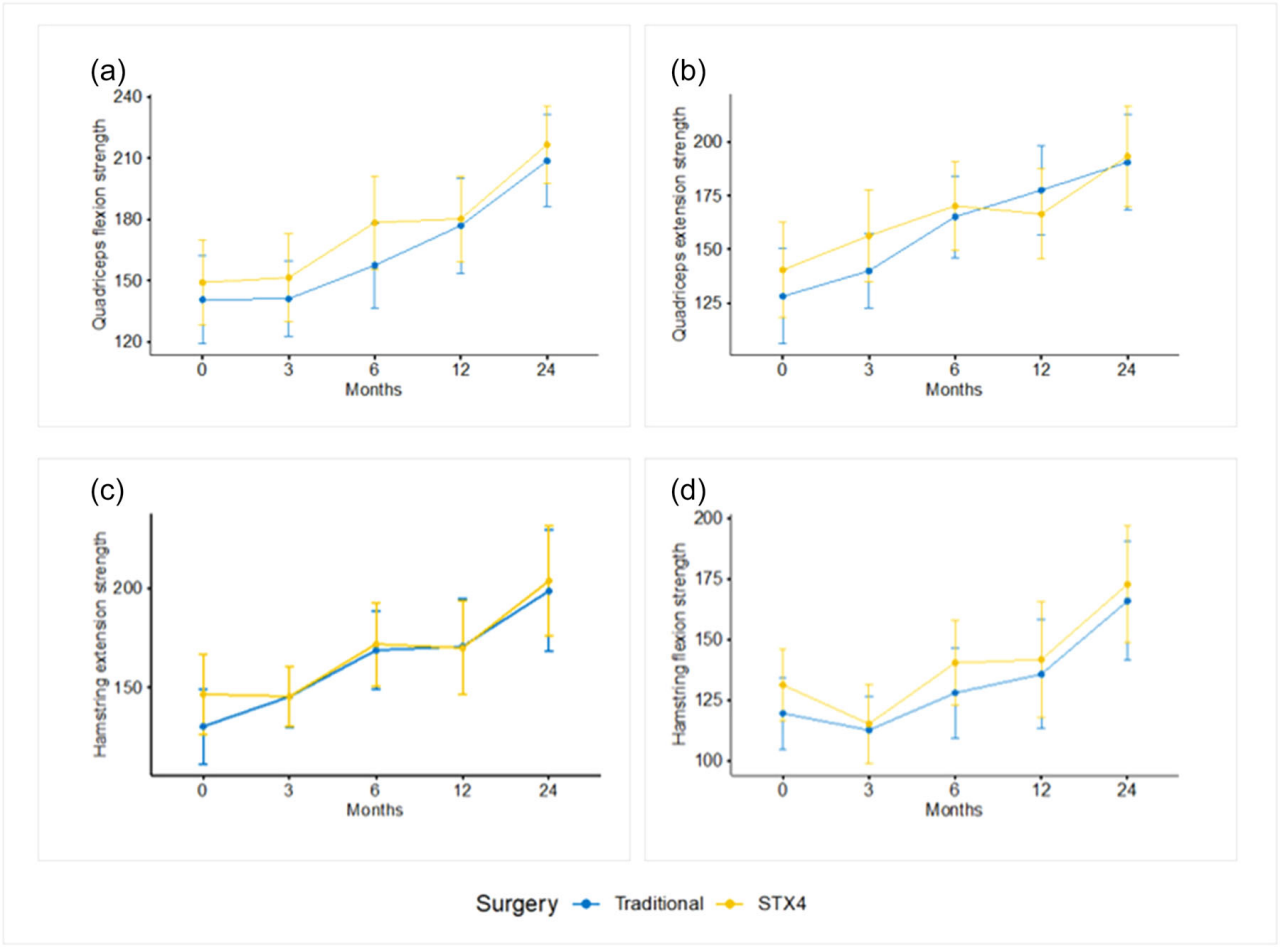
Quadriceps and Hamstrings strength in newtons through time in both groups (a) quadriceps strength in flexion. (b) Quadriceps strength in extension. (c) Hamstrings strength in extension. (d) Hamstrings strength in flexion.

When analysing re‐tear rates, the control group had 2/21 cases (9.5%), and the experimental group had 1/21 case (4.8%), *p* = 0.9. There was one re‐intervention in each group, a case of ACL revision for the control group and a meniscectomy in the experimental group. There were no other complications or adverse events. Variations in PROMs and in strength can be graphically seen in Figures 2 and 3. Return to sport rates at 2 years were 90% (*n* = 19) for the STx4 group and 81% (*n* = 17) for the ST‐G group, with no statistically significant differences (*p* = 0.37). In summary, at 2‐year follow‐up, there were no statistically significant difference in PROMs, muscle strength, anterior tibial translation, return to sport or adverse events.

**Figure 4:**
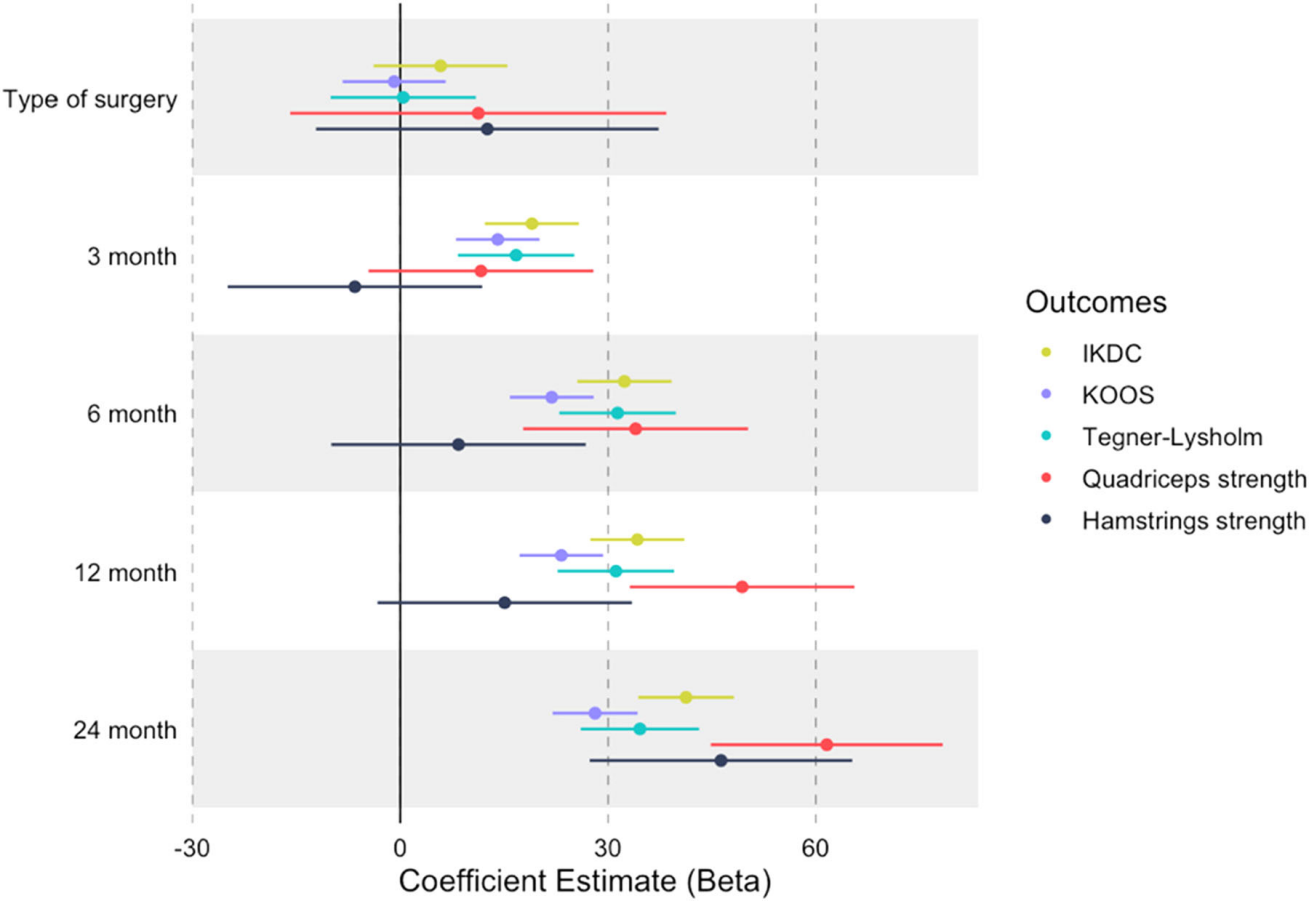
Linear mixed model results for groups comparation and outcomes of interest. IKDC, International Knee Documentation Committee; KOOS, knee injury and osteoarthritis outcome score.

The linear mixed model analysis revealed substantial between‐subject heterogeneity, particularly in muscle strength measurements (σ² = 923.43 for hamstring flexion and 720.74 for quadriceps extension) compared to functional scales (σ² ranging from 99.92 to 194.12). The intraclass correlation coefficients (ICC) demonstrated higher reliability in strength measurements (0.45–0.64) than functional scales (0.34–0.50), validating our longitudinal measurement approach. In terms of clinical outcomes, both muscle strength and functional recovery showed favourable and sustained improvements over time, regardless of surgical procedure type. Specifically, hamstring strength increased significantly at 24 months postsurgery (estimate: 36.40 N, CI: 27.23–45.57, *p* < 0.001), while quadriceps strength recovered from 6 months postsurgery (estimate: 40.11 N, CI: 30.82–49.39, *p* < 0.001), with no significant influence of surgery type (*p* > 0.05), model results for every outcome can be seen in Figure 4. The model's explanatory power, reflected in marginal (0.171–0.460) and conditional (0.546–0.732) *R*² values, supports the importance of including random effects in capturing individual response patterns.

## DISCUSSION

The most important finding of our study is that there were no differences in strength between the quadrupled semitendinosus graft and the semitendinosus‐gracilis graft for ACLr; the sample size may have been underpowered to detect small differences in strength, but the mean strength difference between the groups was not clinically significant. The linear mixed model analysis indicated that strength is recovers more quickly for the quadriceps (6 months) compared to the hamstrings (24 months) in both groups.

At the 12‐month follow‐up, the STx4 group exhibited significantly greater anterior tibial translation compared to the ST‐G group. However, this difference was not statistically significant at the 24‐month time point. The authors are uncertain about the possible explanations for this observation. It may be attributed to chance, measurement error, or the possibility that the grafts continue to remodel for up to 2 years after surgery, reaching their mature state [23]. Cavaignac et al. showed that at 1‐year follow‐up, incorporation and ligamentization of the ST‐G and STx4 grafts were the same based on MRI analysis, despite having shorter tunnels for the STx4 group [7]. Adding a lateral extraarticular tenodesis has shown, better graft maturity in MRI at 1‐year, for STx4 grafts [8]. However, graft maturation has not shown to correlate with PROMs and anterior knee stability [18].

In terms of hamstrings strength, there are mixed results comparing the ST‐G and STx4 grafts in the literature; similarly to the present study, Roger et al. found no statistically significant differences between these type of grafts, in a randomised controlled trial involving 60 patients [29]. However, Kyung et al. found peak torque deficit in flexion being significantly lower in the STx4 group compared to the ST‐G group at 60 and 180 degrees [16]. In a similar way, Kouloumentas et al. reported superiority for the STx4 when compared to the ST‐G in regards to strength [13]. Other reports in literature such as Sengoku's further support these findings [30]. This was also reported by Kulinski et al. after 4.5‐year follow‐up, in which they reported a significant decrease in isokinetic peak torque for flexion in the ST‐G group compared to the contralateral limb [15]. It is important to note that these studies in which there was a difference among groups had significantly larger sample sizes (Kyung: *n* = 144, Kouloumentas: *n* = 90, Sengoku: *n* = 82, Kulinski: *n* = 162) than our study (*n* = 42) and the one performed by Roger et al. (*n* = 60), which might have resulted in underpowered results. Of note, a meta‐analysis by Chin et al. investigating differences between the quadrupled semitendinosus graft and semitendinosus/gracilis graft configuration reported no significant differences in side‐to‐side deficit in isokinetic peak torque between both groups specifically for flexion at 60 degrees [9].

Further research should be performed to investigate the differences in strength among these grafts with larger sample size and longer follow‐up time, as it is biomechanically feasible and logical to consider the possibility that preserving the gracilis would result in increased strength. Besides, sparing the gracilis tendon do have other advantages that are not evident in a clinical trial such as having an extra graft that may be used to reconstruct other ligaments; for example, in associated multiligamentary injuries (for a medial collateral ligament, a medial patellofemoral ligament, etc.) or for a lateral extra‐articular tenodesis procedure, which decreases the risk of ACL re‐tear in high risk patients [28].

As a difference with other publications, most previous studies used an all‐inside technique, while only this study and Kulinski et al. [15] used a femoral cortical button and a tibial interference screw. This is a cheaper technique than an all‐inside technique with equivalent results which can be a safe alternative according to the context of clinical practice. When analysing complications and re‐tear rates, we found no statistically significant differences among groups with only one case of re‐tear occurring in the STx4 group and two in the ST‐G group. In a cadaveric study, investigating the biomechanical properties of different ACL graft configurations, there was a significantly higher tensile strength noted for the quadrupled semitendinosus configuration, with the highest load to failure values and a slightly higher stiffness noted among different graft options [26]. That study concluded that a quadrupled semitendinosus graft would be their graft of choice due to its biomechanical properties, with the gracilis being considered a good option to augment the graft when necessary. Further, a case series study investigating outcomes of 200 patients with ACLr using both semitendinosus and gracilis grafts reported a re‐tear rate of 1.5% at a minimum follow‐up of 1 year [10]. Similar to our findings, Kouloumentas et al. compared STx4 versus ST‐G in ACLr at 2 years and reported failure rates of 2.2% and 4.4% for the quadrupled semitendinosus and the semitendinosus/gracilis groups respectively [13]. Finally, a study by Mo et al. comparing quadrupled semitendinosus versus double‐stranded semitendinosus and gracilis autograft in ACLr found no difference in failure rates between both groups [25]. Most of these studies, including ours, have had rather short follow‐up times, and it would be valuable to investigate failure and revision rates in cohorts with longer follow‐up times.

We investigated PROMs in both groups and found no difference in most scores except for the Lysholm Tegner in which the STx4 group had a favourable outcome at 3‐month follow‐up. Interestingly, mixed evidence regarding differences in PROMs when comparing these graft configuration types exists. Kulinski et al. found no statistically significant difference between groups for the baseline pain and functional outcomes (visual analogue score, KOOS pain, IKDC, Lysholm and Tegner). However, they found a statistically significant difference in the percentage of patients achieving the patient acceptable symptom state (PASS) for the KOOS pain subscale, favouring the ST‐G group [15]. Our findings of comparable outcomes in PROMs are further supported by Kouloumentas et al. who reported no difference in IKDC, KOOS and knee society score (KSS) at 2 years, and Mo et al., in which there were also no differences in IKDC or KOOS among the two types of graft configurations analysed in this study at 2 years follow‐up [13, 25]. On the other hand, Kyung reported no differences in the Lysholm Tegner scale, for which we found a statistically significant difference between the two graft types studied at a certain point [16]. Further, when comparing return to sport rates we found no statistically significant difference between groups. Our findings align with those reported by Kulinski et al., in which no difference in return to sport rates was found at 4.5 years follow‐up comparing the same graft configurations and type of fixation we studied [15]. Our study population was heterogenous with regards to activity performance types, it would be interesting for further studies to assess how the use of either graft type can impact return to sport looking at high‐level athlete cohorts.

Overall, our findings show both graft configuration types to have no statistically significant differences at 2 years regarding the different outcomes, including strength, PROMs, return to sport and adverse events. Hence, it is clinically important for sports surgeons to be aware of these findings when assessing patients in which sparing the gracilis is desirable or when the gracilis is not available, such as in multiligament injury scenario (where the gracilis could be useful to reconstruct another ligament), in patients with previous surgery in which the gracilis has already been harvested, or when the gracilis is damaged during the harvesting process. On the other hand, utilising both tendons can be advantageous in the case of a quadrupled semitendinosus graft with an insufficient diameter (<8 mm), as the gracilis tendon can help increase the overall diameter.

### Limitations

Our study has some limitations, such as the sample size which might have caused our results to be underpowered and might be the reason to why we did not find any differences in knee strength among groups. Additionally, the 2‐year follow‐up could be insufficient for some outcomes, such as re‐tears or additional surgeries; though, it was enough time to evaluate our primary outcome (strength). Finally, our surgical technique was performed using a closed tendon stripper and tendons were detached from the tibia, in such a way that there is a risk of partially affecting the gracilis insertion when harvesting only the semitendinosus. This could have the potential to bias our results for some patients, underestimating the hamstrings strength in the experimental group.

## CONCLUSIONS

This study found no statistically significant differences at 2‐years between a STx4 and a ST‐G graft configuration for primary ACLr regarding strength, PROMs, return to sport, failure rates and complications; larger studies are required to confirm noninferiority. Both graft options are safe and can be considered viable options when performing this type of procedure. As the gracilis tendon might be useful for reconstructing additional structures, sparing it and harvesting only the semitendinosus is a good alternative.

## AUTHOR CONTRIBUTIONS

**Juan P. Martinez‐Cano**: Original idea; study design; analysis; writing and editing the draft. **Alejandro Gallego, Janio Cuadros** and **Laura Ibarra**: Data collection; analysis; writing of the draft. **María V. Velasquez‐Hammerle**: Analysis and editing the draft. **Fernando M. Mejía** and **Alfredo Martinez‐Rondanelli**: Study design; writing of the draft.

## CONFLICT OF INTEREST STATEMENT

The authors declare no conflicts of interest.

## ETHICS STATEMENT

IRB: Comité de Ética en Investigación Biomédica from Fundación Valle del Lili. Approval number: 1183. Written consent was obtained from all patients.

## Supporting information


**Supplementary file 1**. Including figures regarding the surgical technique.

## Data Availability

The data that support the findings of this study are available on request from the corresponding author. The data are not publicly available due to privacy or ethical restrictions.
