# Supplementary material for: Similar results with quadrupled semitendinosus and semitendinosus‐gracilis graft in anterior cruciate ligament reconstruction: A randomised controlled trial with 2‐year follow‐up
Source: J Exp Orthop. 2025 Sep 24;12(3):e70399. doi: 10.1002/jeo2.70399 (PMC12460705; doi:10.1002/jeo2.70399)

Supplementary file 1.

**Figure I.** Quadrupled semitendinosus graft with cortical button in one edge and sutures in the other edge. The minimum length should be 60 mm.

**Figure II.** Figure IIA, shows the incision mark at the pes anserinus insertion in the medial tibia. Figure IIB, the pes anserinus fascia is identified. Figure IIC, the semitendinosus tendon is identified and extracted. Figure IID, the semitendinosus tendon is measured and ready to be prepared.


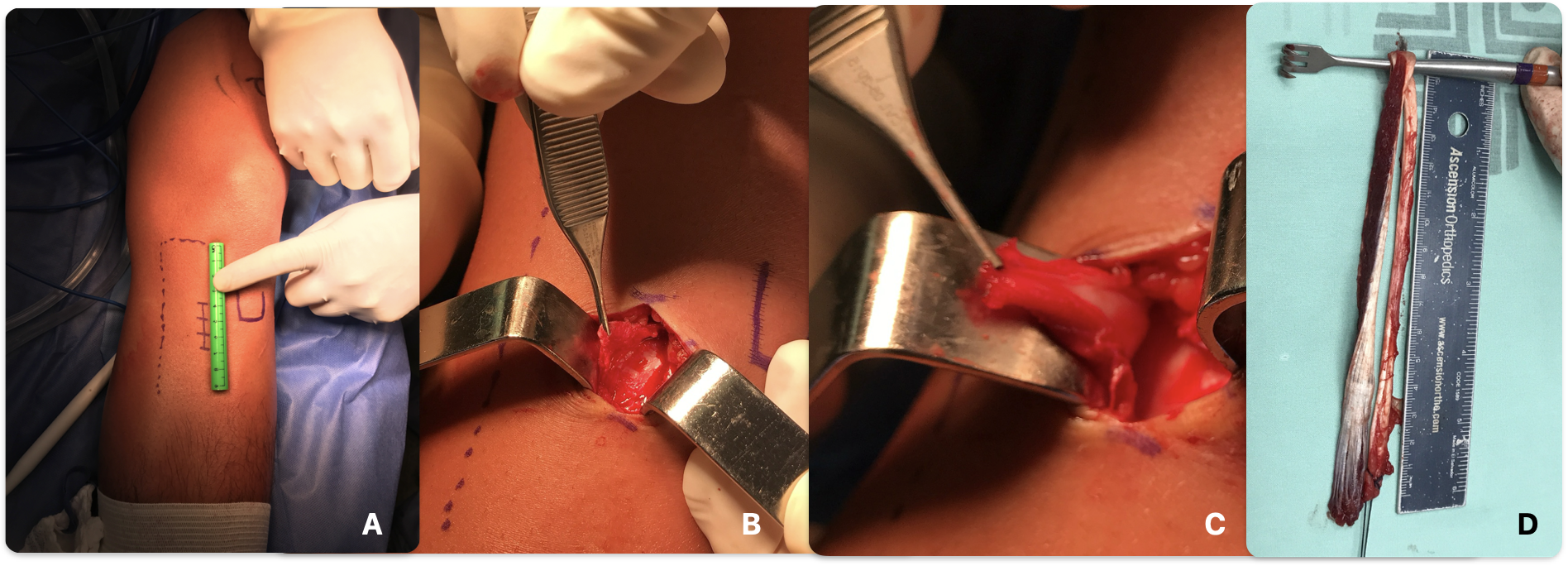


**Figure III.** Quadrupled semitendinosus final disposition in the anterior cruciate ligament reconstruction with cortical button in femur and interference screw in tibia.


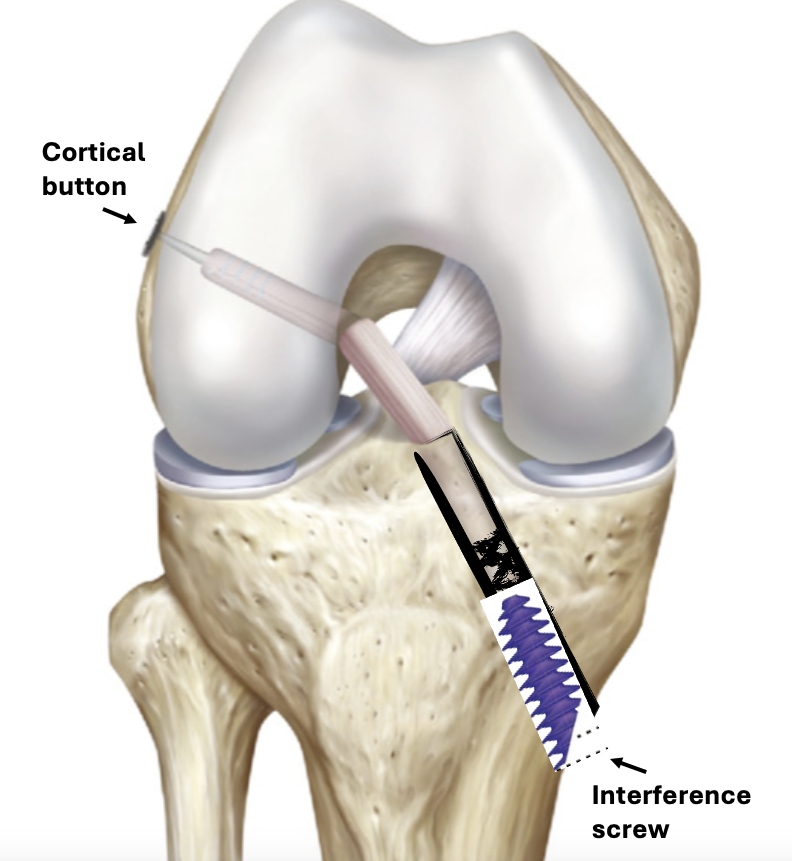

Supplement: Supplementary file 1 — Including figures regarding the surgical technique. [file JEO2-12-e70399-s001.docx]
